# Design and Optimization of a Twisted Photodiode Pixel Structure for All-Directional Phase-Detection Autofocus CMOS Image Sensors †

**DOI:** 10.3390/s26061758

**Published:** 2026-03-10

**Authors:** Daiki Shirahige, Koichi Fukuda, Hajime Ikeda, Yusuke Onuki, Ginjiro Toyoguchi, Kohei Okamoto, Shunichi Wakashima, Hiroshi Sekine, Shuhei Hayashi, Ryo Yoshida, Junji Iwata, Yasushi Matsuno, Katsuhito Sakurai, Hiroshi Yuzurihara, Takeshi Ichikawa

**Affiliations:** 1 Device Technology Development Headquarters, Canon Inc., Kawasaki 212-8602, Japan; 2 Graduate School of Engineering, Tohoku University, Sendai 980-8577, Japan; 3 Imaging Business Operations, Canon Inc., Tokyo 146-8501, Japan

**Keywords:** CMOS image sensor, phase detection autofocus, twisted photodiode, charge transfer, all-directional AF

## Abstract

To achieve an all-directional and high-speed, high-accuracy autofocus (AF) function, we propose a CMOS image sensor with a Twisted Photodiode (PD) structure. The developed 3D-stacked back-side illuminated (BSI) sensor employs the Twisted PD, which enables equivalent angular response characteristics in both the horizontal and vertical directions for the two PDs integrated within a single pixel, thereby realizing AF detection for all pixels and all directions. This paper describes the Twisted PD structure that enables all-directional AF and presents an analysis of charge transfer behavior in this unique 3D configuration. In this paper, “all-directional” refers to robustness with respect to subject direction.

## 1. Introduction

CMOS image sensors (CIS) are widely used in applications such as mobile devices, digital still cameras, automotive systems, and security, where continuous improvements in both image quality and functionality are required. Among these functions, autofocus (AF) is one of the most critical features, particularly in demanding scenes requiring fast and accurate focusing performance. To achieve higher AF speed and robustness, various phase detection autofocus (PDAF) pixel structures have been proposed [1,2,3].

In conventional horizontally split dual-pixel structures, phase detection can be performed only for vertical line patterns, whereas horizontal line patterns cannot be detected due to the lack of phase difference along that direction. Therefore, realizing all-directional phase detection has become an important requirement for advanced PDAF sensors. However, enabling all-directional phase detection generally introduces structural complexity and often compromises pixel symmetry, which can negatively impact image quality.

Several approaches have been reported to address this limitation. For example, the Quad PD structure enables phase detection in both horizontal and vertical directions, but it increases the number of photodiodes per pixel, resulting in longer readout time, increased floating diffusion (FD) capacitance, and higher dark random noise (DRN). The Slanted PD structure achieves all-directional phase detection by arranging adjacent pixels diagonally [4], but it suffers from reduced detection accuracy and requires additional signal processing. The Rotated PD structure realizes cross-type detection by rotating pixel orientation [5,6]; however, mismatches in transistor layout and interconnections can lead to FD capacitance imbalance and potential degradation of photo-response non-uniformity (PRNU).

These prior studies indicate that achieving all-directional phase detection is not merely a matter of increasing directional sensitivity, but rather involves a fundamental structural trade-off between phase-detection capability and pixel-level symmetry. In particular, multidirectional phase detection often requires asymmetrical pixel layouts or orientation-dependent structures, which can intrinsically degrade electrical balance and image quality. Therefore, the challenge lies in realizing all-directional phase detection while preserving structural equivalence and electrical robustness across pixel orientations. In other words, an ideal PDAF pixel should satisfy the following fundamental design requirements derived from these structural considerations:

(i) all-directional phase-detection capability,
(ii) minimal structural mismatch between pixel orientations, and
(iii) stable electrical characteristics that preserve image quality.

In this context, we propose a Twisted Photodiode (Twisted PD) structure as a structure-driven design concept that mitigates this fundamental trade-off. By decoupling the phase-detection directionality between the front-side and back-side layers while maintaining equivalent in-plane pixel geometry, the proposed structure enables all-directional phase detection without sacrificing pixel-level symmetry. In this configuration, a vertical pixel is placed at the Gb pixel position and rotated by 90 degrees between the front-side and back-side layers [7]. The back side adopts a vertical-split configuration enabling horizontal phase detection, while the front side retains the same horizontal-split configuration as the conventional horizontal pixel. This design significantly reduces structural differences between horizontal and vertical pixels, thereby improving robustness against orientation-dependent process variations. Imaging experiments with a sensor equipped with the Twisted PD have confirmed improved AF accuracy [8].

While previous work mainly demonstrated pixel architecture and phase-detection operation, a critical open question remains as to whether the proposed three-dimensional structural concept is physically compatible with efficient charge transport. In other words, reliable and fast charge transfer is not only a requirement for practical implementation, but also a necessary condition to validate the underlying structural design principle. Several studies have analyzed charge transfer characteristics in conventional pixel structures to shorten transfer time through structural optimization [9]. In the Twisted PD, photo-generated charge from the back side must pass through a slit toward the charge storage region on the front side, resulting in a more complex 3D transfer path compared with conventional designs. Therefore, it is essential to verify that the electrostatic potential distribution supports efficient charge transfer without introducing delay or degradation.

In this study, we analyze the charge transfer behavior in the Twisted PD structure through device simulations of electrostatic potential distribution and transient charge transport dynamics. This analysis serves as a physics-based validation of the proposed structural concept, confirming that the three-dimensional twisted configuration does not introduce adverse potential barriers or delay in charge transfer. The results demonstrate that the designed electrostatic potential enables fast and reliable charge transfer even in the presence of the intricate 3D transport path. Although a detailed quantitative evaluation of electrical parameter mismatch is beyond the scope of this manuscript, this work provides important structural design considerations for reducing sensitivity to orientation-dependent process variations.

This manuscript is based on the same test chip previously reported at IEDM 2023 [7]. While the IEDM 2023 paper primarily focused on pixel architecture and basic phase-detection operation, the present work places emphasis on charge transfer mechanisms and device-level characteristics of the Twisted PD structure. Table 1 provides a qualitative comparison of different PDAF pixel structures to highlight general performance tendencies and design trade-offs.

## 2. Sensor Architecture

The twisted photodiode structure is designed to maintain structural symmetry between horizontal and vertical phase-detection pixels, enabling all-directional phase-detection operation without introducing pixel-dependent imbalance. Such symmetry is essential for ensuring consistent phase-detection behavior across different pixel orientations, particularly in advanced PDAF image sensors. From a manufacturing perspective, minimizing structural differences between pixel orientations is also important for reducing orientation-dependent sensitivity to lithography and implantation variations. However, introducing a twisted photodiode geometry inevitably modifies the internal electrostatic potential distribution and carrier transport paths. Therefore, a detailed charge transport analysis is required to confirm that the proposed structure maintains reliable charge transfer behavior despite its three-dimensional geometry. This analysis serves as a first verification that the twisted geometry does not compromise the fundamental requirement of fast and complete charge transfer in PDAF pixels.

**Figure 1:**
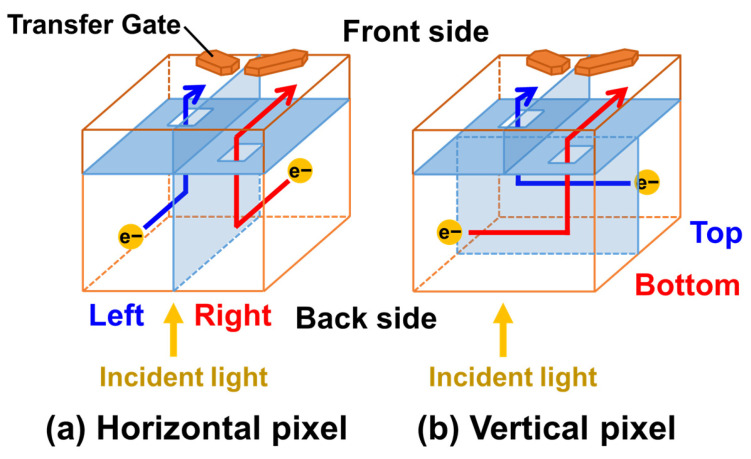
Charge transfer paths.

Figure 1 illustrates the charge transfer paths within the proposed pixel. Each pixel is divided into two photodiodes (PDs) by a PN junction to enable phase detection autofocus (PDAF). Furthermore, each PD is also separated into front-side and back-side regions by another PN junction. A slit is formed in part of this PN junction, through which the depletion region extends toward the back side. Photo-generated charges created in the back- side region move toward the front-side charge storage region along the potential gradient.

**Figure 2:**
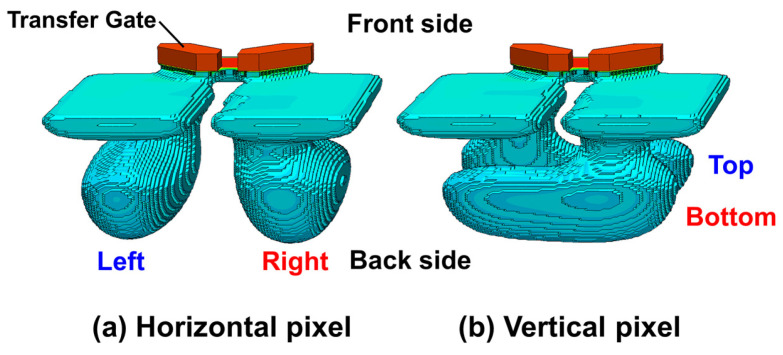
3D TCAD simulation results for potential profiles.

Figure 2 shows the simulated 3D potential profile of the pixel. For both horizontal and vertical pixels, a monotonic potential distribution is formed from the back side to the front side, enabling efficient charge transfer without introducing orientation-dependent degradation. In the Vertical pixel, the potential configuration is designed so that the PD split direction on the front side and back side are rotated by 90°, thereby enabling phase detection for horizontal line patterns of the object.

**Figure 3:**
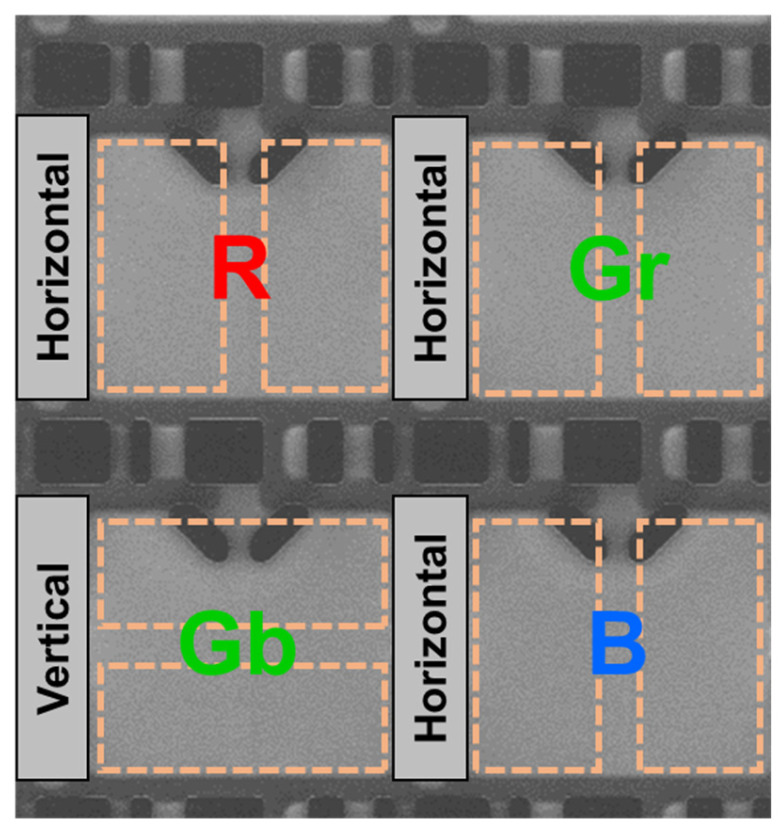
Top-view SEM image of pixel layout showing horizontal and vertical pixels.

Figure 3 shows a top-view scanning electron microscope (SEM) image of the pixel layout. Vertical pixels are placed at the Gb positions in the Bayer array, while horizontal pixels are located at the R, Gr, and B positions. As shown in Figure 3, the shallow trench isolation (STI) and polysilicon patterns on the silicon surface are identical between horizontal and vertical pixels. If the vertical pixel were designed by rotating all STI, polysilicon, and metal wiring layers by 90°, device-to-device variations would arise due to structural differences from the horizontal pixels. Such full rotation would alter the relative placement and routing length of readout-related components and interconnects, increasing the risk of orientation-dependent electrical variations. In contrast, the Twisted PD structure achieves all-directional PDAF while preserving identical surface-level circuitry, thereby reducing orientation-dependent electrical mismatch.

**Figure 4:**
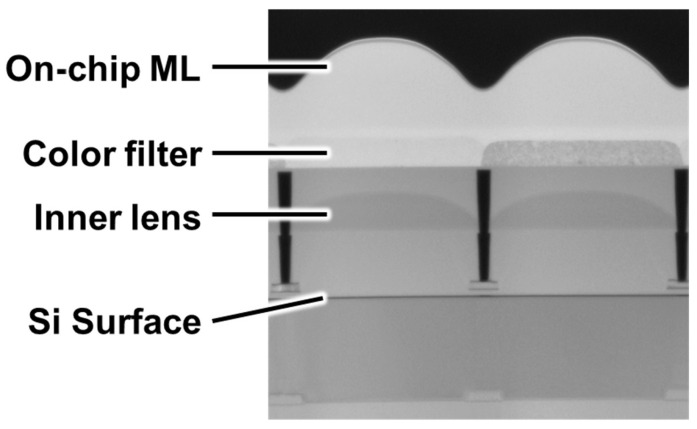
Cross-sectional TEM image of the pixel structure.

**Figure 5:**
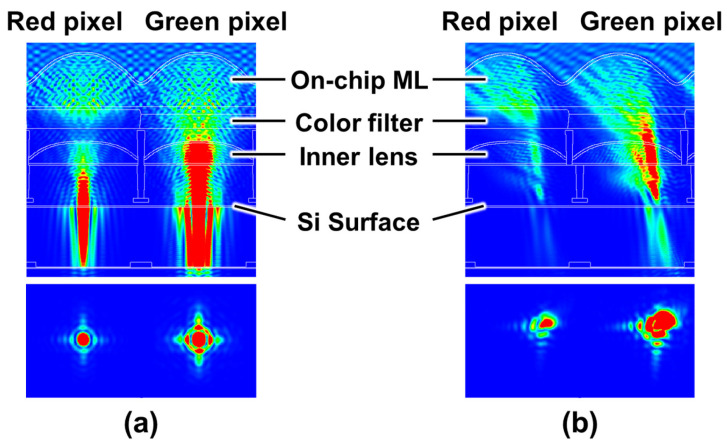
Optical simulation results for (**a**) pixels at the sensor center and (**b**) pixels at the sensor edge. The color map represents the optical intensity (blue: low, red: high).

Figure 4 shows a cross-sectional transmission electron microscope (TEM) image of the pixel, and Figure 5 presents optical simulation results at the center and edge of the sensor. Figure 4 and Figure 5 describe the optical configuration of the proposed pixel, which is introduced to establish realistic illumination conditions for the subsequent device simulations. In particular, the optical simulations are used to determine representative light incidence positions under both on-axis and peripheral-ray conditions. The developed sensor employs a stacked optical structure consisting of an inner microlens (IML), color filter, and microlens above the photodiode. To enhance sensitivity and suppress optical crosstalk, various IML technologies have been proposed in previous studies [10,11]. Yamada et al. [10] reported that placing a microlens directly above the photo-electric conversion region in CCD image sensors improves both light collection efficiency and smear characteristics by increasing the effective pixel aperture. Subsequently, Takahashi et al. [11] introduced the IML structure into CMOS image sensors, demonstrating efficient light focusing and improved sensitivity. These studies indicate that the IML is widely regarded as an important technology for maintaining optical efficiency as pixel sizes continue to shrink. In this study, an IML is implemented to focus incident light onto each photodiode, forming a pixel optical structure consistent with those reported in prior studies. In addition, an optical isolation structure is introduced between adjacent IMLs. Several optical isolation techniques have been proposed to improve pixel performance. Park et al. [12] reported a non-metallic pixel isolation structure that effectively suppresses optical crosstalk and enhances sensitivity in submicron CMOS pixels. Skorka et al. [13] proposed a quantitative method to evaluate chromatic flare artifacts, highlighting the importance of stray-light suppression in image sensors. In the developed pixel, a similar optical separation structure between the IMLs is employed, following the design concepts reported in these studies. To accommodate oblique incident light at the sensor periphery, a pixel-by-pixel shift of the microlens and color filter positions is applied. The optical background of this technique was analyzed by Kuo [14], and implementation optimization was reported by Hwang [15]. A similar lens-shift structure is adopted in this study to account for oblique incident light. Based on three-dimensional finite-difference time-domain (3D-FDTD) optical simulations, the incident light positions at both the center and the edge of the sensor were analyzed. For edge pixels, simulations were conducted under the peripheral ray incidence condition corresponding to an F-number of 1.8. It was confirmed that light enters regions displaced from the pixel center due to the large incident angle. These optical simulation results define the possible range of charge-generation locations within the pixel, and therefore provide the essential input conditions for the subsequent electrostatic potential and charge-transport evaluations. It should be noted that the inner microlens and optical isolation structures are described here to establish realistic optical boundary conditions for the simulations. In this study, these optical structures are not quantitatively evaluated as independent performance-enhancing elements; instead, the optical simulations are used to determine representative light incidence conditions for the subsequent charge transport simulations.

**Figure 6:**
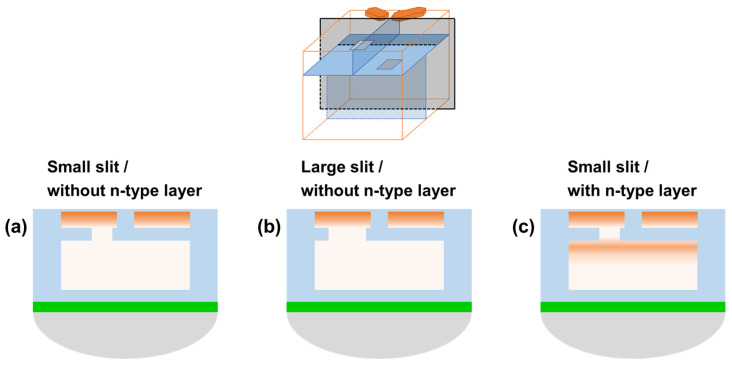
Cross-sectional view of the top PD in the Twisted PD pixel structure. (**a**) small slit without n-type layer, (**b**) large slit without n-type layer, (**c**) small slit with n-type layer.

Figure 6 shows the pixel structures investigated for the Twisted PD. Based on the structure shown in Figure 6a, we examined two variations: Figure 6b a structure in which the slit area in the p-type layer separating the front-side and back-side regions was enlarged, and Figure 6c a structure in which an n-type layer was additionally introduced beneath the separating p-type layer. To ensure reliable PDAF operation, the Twisted PD must establish a sufficient electrostatic potential gradient from the back side toward the front-side charge storage region. Therefore, we investigated several structural variations aimed at improving depletion extension and charge transfer on the back side, as summarized in Figure 6. The additional n-type layer introduced in this pixel structure is a single n-type layer, and no multiple n-type layers are employed. A distinctive feature of the Twisted PD is that a slit is formed in part of the p-type layer that separates the front-side and back-side regions. This configuration enables the formation of either horizontal or vertical pixels simply by changing the orientation of the separating layer between the back side photodiodes. Furthermore, by keeping the slit position and the back side PD separation layer identical between the horizontal and vertical pixels, the design minimizes potential variations and process-induced differences between the two pixel types. However, the presence of the p-type layer separating the front-side and back-side regions can hinder the depletion layer from extending from the front side toward the back side, raising concern that an adequate electric field may not be formed toward the front side where charge is accumulated. To address this issue, we investigated the structures shown in Figure 6, which are designed to establish an appropriate potential gradient extending to the back-side region. In these structures, enlarging the slit opening is expected to increase the likelihood of residual charge remaining after transfer; therefore, it is necessary to limit the slit size to a range where it does not cause transfer residue. Accordingly, in this work, we do not regard the large-slit structure as the primary subject of detailed investigation. Instead, we focus mainly on the configuration that combines a small slit with an additional n-type layer.

**Figure 7:**
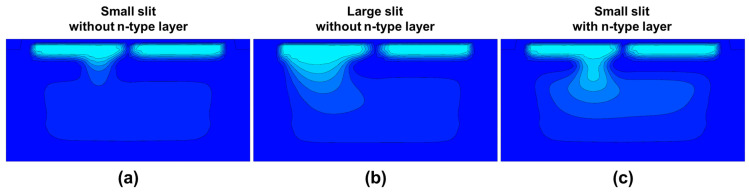
Simulated potential profiles of the top PD for the three structural variations. (**a**) small slit without n-type layer, (**b**) large slit without n-type layer, (**c**) small slit with n-type layer. The color map represents the simulated potential distribution, where darker blue indicates higher potential and lighter blue indicates lower potential for electrons.

Figure 7 presents the simulated potential profiles of the three pixel structures described in Figure 6. In the original structure before slit enlargement (Figure 7a), the p-type layer separating the front-side and back-side regions suppresses depletion extension toward the back side, resulting in a weak potential gradient from the back side toward the front side. As a consequence, photo-generated electrons in the back-side region are not efficiently guided toward the intended front-side collection node, which can increase undesired charge migration between the paired sub-photodiodes. In contrast, the enlarged-slit structure (Figure 7b) allows the depletion layer to extend through the widened opening, forming a stronger potential gradient even in the back-side region. Although this approach is effective for improving the electric field distribution, it may increase the risk of residual charge after transfer, as discussed above. Furthermore, in the structure with an additional n-type layer beneath the separating p-type layer (Figure 7c), a more favorable back-side potential profile is achieved without enlarging the slit size. This optimized potential gradient enhances charge transfer toward the front side while simultaneously suppressing charge crosstalk between the two sub-photodiodes.

**Figure 8:**
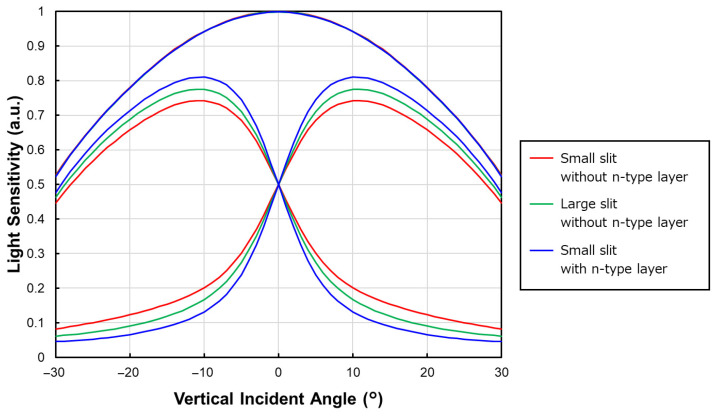
Light sensitivity dependence on incident angle for three pixel structures: small slit without n-type layer, large slit without n-type layer, and small slit with n-type layer.

Such back-side potential optimization is particularly important for PDAF operation because phase detection relies on generating a sufficient signal difference (phase-detection contrast) between the paired sub-photodiodes under oblique illumination. Accordingly, angular-response measurements are widely used as a key indicator of PDAF performance in prior implementations [16,17]. At a given incident angle, a larger difference in the output signals of the two sub-PDs indicates stronger charge separation and reduced crosstalk, resulting in higher phase-detection contrast. To experimentally verify that the improved back-side potential directly enhances PDAF functionality, we measured the angular responses of the prototype chip. As shown in Figure 8, both the enlarged-slit and n-type-inserted structures exhibit larger angular-response separation than the original structure, consistent with the TCAD-predicted potential gradients. This confirms that an optimized back-side potential profile effectively improves charge separation between paired sub-PDs.

**Figure 9:**
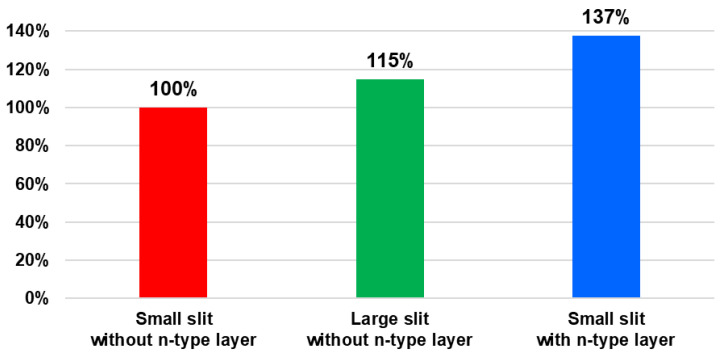
Normalized baseline lengths for three pixel structures: small slit without n-type layer, large slit without n-type layer, and small slit with n-type layer.

Building on these angular-response results, Figure 9 further quantifies the improvement in vertical-direction PDAF sensitivity using the baseline length, which represents the effective pupil-separation distance. Under a practical small-aperture condition (F/16), the centroid angle (the sensitivity-weighted mean incident angle) was calculated for each sub-photodiode within the incident-angle range defined by the F/16 cone. The baseline length was then defined as the absolute difference between the centroid angles of the left and right sub-PDs [18]. The normalized results in Figure 9 show that introducing the n-type layer on the backside provides a 32% increase in baseline length compared with the reference structure. This improvement directly reflects the enhanced phase-detection contrast achieved through back-side-potential optimization, leading to more robust phase-detection performance in the Twisted PD architecture.

**Figure 10:**
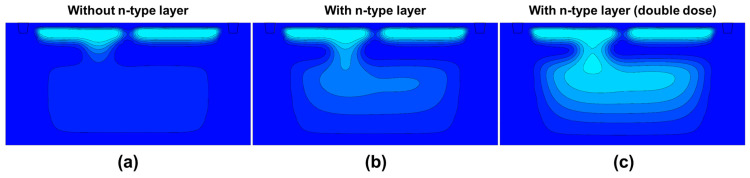
Simulated potential profiles of the top PD for the three n-type layer configurations. (**a**) without n-type layer, (**b**) with n-type layer, (**c**) with n-type layer (double dose). The color map represents the simulated potential distribution, where darker blue indicates higher potential and lighter blue indicates lower potential for electrons.

To further investigate the effect of the n-type implantation dose, device simulations were conducted with varying n-type impurity concentrations while keeping the slit geometry unchanged. Figure 10 compares the simulated potential profiles across the slit region of the top PD for three cases: (a) without an n-type layer, (b) with an n-type layer, and (c) with an n-type layer at double dose. The introduction of the n-type layer clearly strengthens the back-side-to-front-side potential gradient, thereby providing a stronger driving field for electron transport through the slit.

**Figure 11:**
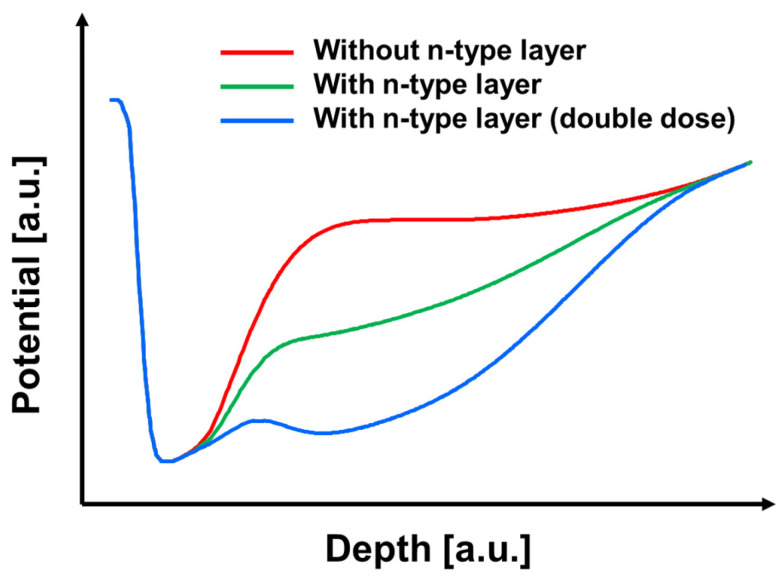
Depth-direction potential profiles extracted along the slit region for three configurations: without an n-type layer, with an n-type layer, and with an n-type layer at double doping concentration.

To examine the charge transfer path in more detail, one-dimensional potential profiles were extracted along the vertical transport direction through the slit, as shown in Figure 11. In the case without n-type doping, although an overall potential slope exists from the back-side photosensitive region toward the front-side charge storage region, a partially flat potential segment appears along the transport path. Such a weak-field region is unfavorable because it can slow down drift-driven electron transfer. In contrast, with an appropriate n-type implantation dose, the potential gradient becomes steeper and more continuous, enabling photo-generated electrons to be efficiently guided toward the charge storage region. However, when the n-type dose is doubled, the electron potential becomes excessively lowered in the doped region, forming a potential pocket that can trap electrons and hinder charge transfer. It should be noted that the back-side potential profile is not intended to be perfectly linear over the entire depth direction. Near the front side, a steep potential drop is intentionally required to ensure reliable charge accumulation in the storage region. Meanwhile, in the back-side photosensitive region and along the slit transport path, the potential gradient is optimized to promote drift-assisted electron transfer. Therefore, the proposed potential design represents a practical balance between efficient back-side charge transport and stable front-side charge storage, rather than an idealized linear distribution. Based on these simulation results, an optimal n-type doping concentration was determined from the viewpoint of achieving efficient charge transfer without introducing detrimental potential pockets.

**Figure 12:**
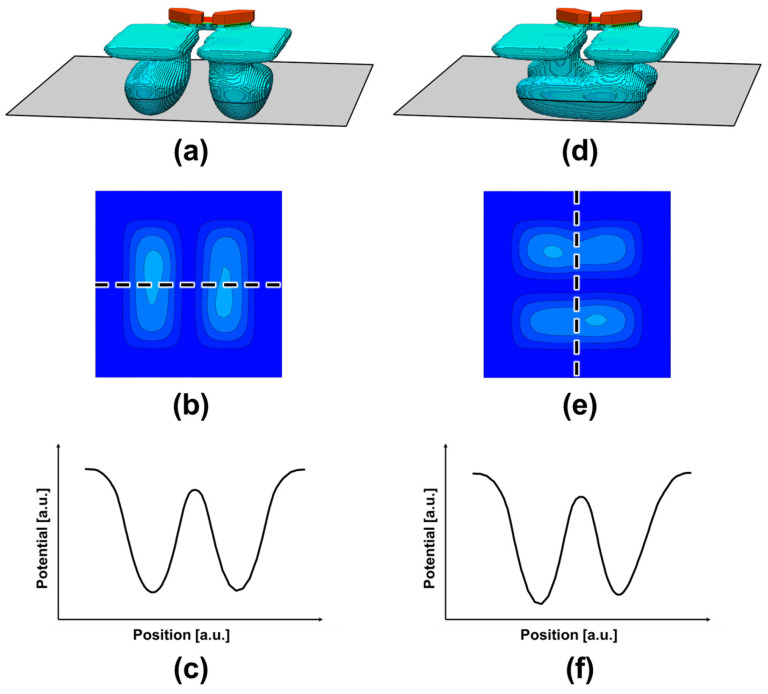
Simulated potential profiles in the back-side photosensitive region for horizontal (**a**–**c**) and vertical (**d**–**f**) pixels: (**a**,**d**) cross-sectional locations; (**b**,**e**) 2D potential maps; (**c**,**f**) 1D profiles showing the potential barrier between adjacent PDs.

Figure 12 summarizes the simulated potential distributions in the back-side photosensitive regions of the designed Twisted PD, where most photoelectric conversion occurs. Because the generated electrons must be transported from the back side toward the intended front-side charge storage region of each sub-photodiode, it is essential to suppress undesired charge migration into the neighboring PD. Such charge leakage would directly increase electrical crosstalk and degrade phase-detection autofocus accuracy. Therefore, even in the three-dimensionally twisted geometry, the pixel must maintain a sufficient potential barrier between the two sub-photodiodes to ensure proper charge separation. As confirmed in Figure 12, both the horizontal and vertical pixel configurations form a clear potential barrier between the paired PDs, indicating that electron confinement is maintained despite the complex transfer paths. This barrier design is a key requirement for achieving robust all-directional PDAF operation in the Twisted PD architecture. It should be noted that the potential profiles in Figure 12f are not perfectly symmetric between the two photodiodes. This asymmetry originates from the proximity of the floating diffusion (FD) region to the Top PD. Since the FD is located in the shallow region to enable charge transfer through the transfer gate, an intentional potential barrier is introduced beneath the FD to prevent direct electron leakage. As a consequence, the Top PD exhibits a locally elevated potential level, as observed in Figure 12e, reflecting controlled charge confinement rather than unintended imbalance.

**Figure 13:**
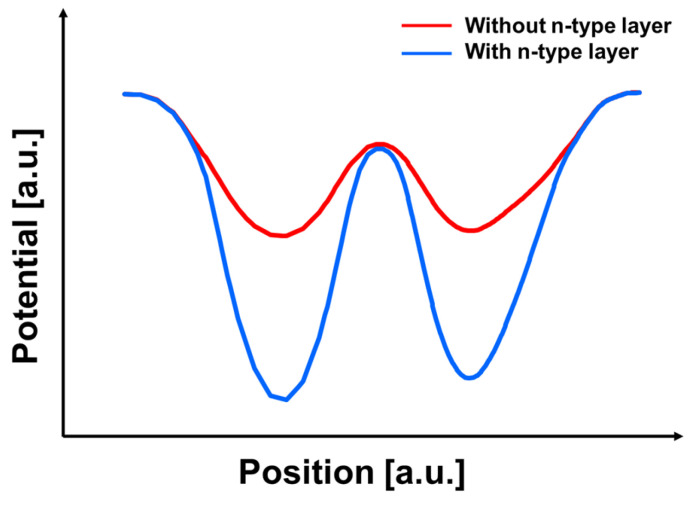
Comparison of potential profiles in the photosensitive region with and without the n-type layer, showing differences in the potential barrier along the charge transfer path.

To further clarify the effect of the additional n-type layer, Figure 13 compares the back-side potential profiles with and without the n-type implantation. Although the n-type layer slightly lowers the nominal inter-PD barrier height, it significantly lowers the potential along the intended electron transport path. As a result, the effective barrier against lateral charge migration is increased, producing a potential configuration that is less prone to charge crosstalk during back-side-to-front-side transfer.

**Figure 14:**
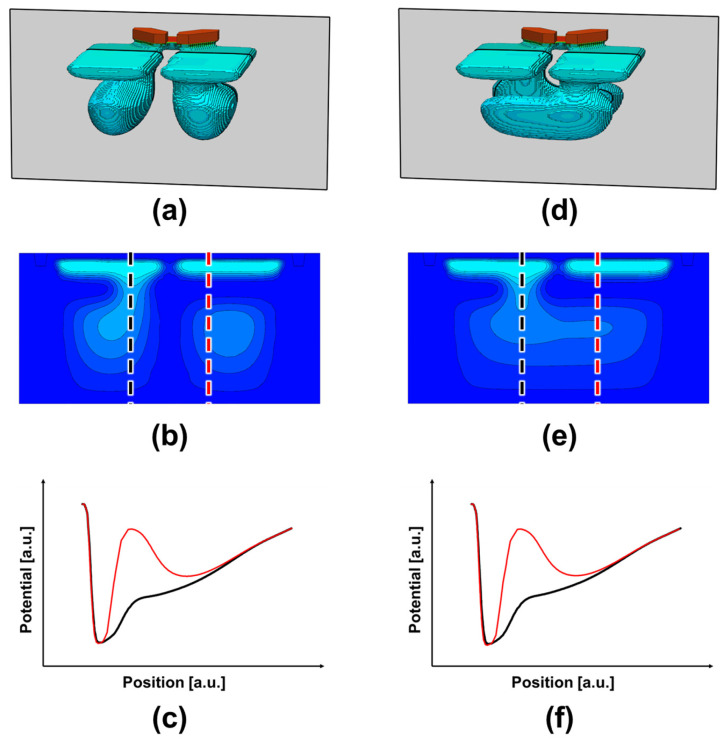
Depth-direction potential profiles at the slit and non-slit regions for horizontal (**a**–**c**) and vertical (**d**–**f**) pixels: (**a**,**d**) cross-sectional locations; (**b**,**e**) 2D potential maps; (**c**,**f**) 1D profiles comparing potential gradients from the back side to the front side. The black and red lines indicate the slit and non-slit regions, respectively.

Finally, Figure 14 examines the depth-wise potential profiles from the front side to the back side at both slit and non-slit regions for horizontal and vertical pixels. At the slit region, a clear back-side-to-front-side potential gradient is formed, enabling efficient drift-assisted electron transfer toward the charge storage node. In contrast, in regions without a slit, appropriate potential barriers remain on both the front side and back side, preventing electrons from being unintentionally transported along undesired paths. These results confirm that the Twisted PD achieves the required combination of (i) strong inter-PD potential barriers for charge separation and (ii) well-defined potential gradients along the slit region for efficient charge transfer. Such controlled potential engineering is essential for minimizing crosstalk while maintaining high-precision all-directional PDAF performance.

**Figure 15:**
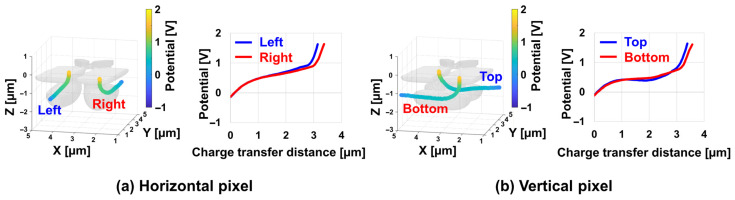
Simulated potential profiles along the charge transfer paths for each PD in the Twisted PD structure.

Figure 15 summarizes the simulated potential variations along the intended charge transport paths for all four sub-photodiodes (top, bottom, left, and right) in both the horizontal and vertical Twisted PD pixels. Because the proposed architecture introduces a three-dimensionally twisted transfer geometry, it is essential to confirm that every sub-pixel maintains a consistent potential gradient that reliably guides photo-generated electrons from the back-side photosensitive region toward the front-side charge storage node. As shown in Figure 15, all four PD types exhibit a monotonic back-side-to-front-side potential slope through the slit region, indicating that the electric field is properly established along each transport path. This confirms that the Twisted PD achieves uniform charge-transfer conditions across different pixel orientations, despite its complex three-dimensional configuration.

In this section, we investigated the electrostatic potential design of the Twisted PD through TCAD device simulations. The results demonstrate that optimization of the slit geometry, together with the introduction of an additional n-type layer, enables (i) well-defined potential gradients for efficient back-side-to-front-side charge transfer and (ii) sufficient inter-PD potential barriers to suppress charge crosstalk between neighboring sub-photodiodes. These simulation findings provide a rigorous physical basis for the proposed pixel design and establish the necessary conditions for robust all-directional PDAF operation. Building on this potential-engineering validation, the next section will present a detailed analysis of the actual charge transport behavior and its experimental verification in the fabricated prototype sensor.

## 3. Results and Discussion

In the previous section, we investigated the potential design of the proposed Twisted PD structure through device simulations and confirmed that appropriate potential gradients and barriers can be formed even along the three-dimensionally complex charge transport paths. Building on these findings, this section evaluates the actual charge transfer behavior of photo-generated carriers in the Twisted PD. Specifically, time-dependent device simulations are performed to verify that charges generated in each back-side photosensitive region are rapidly and reliably transported to the front-side charge storage region without crosstalk. Furthermore, the optical sensitivity and phase-detection performance of both horizontal and vertical pixels are experimentally characterized, demonstrating that the proposed Twisted PD enables all-directional phase detection while maintaining high sensitivity and robust autofocus operation.

**Figure 16:**
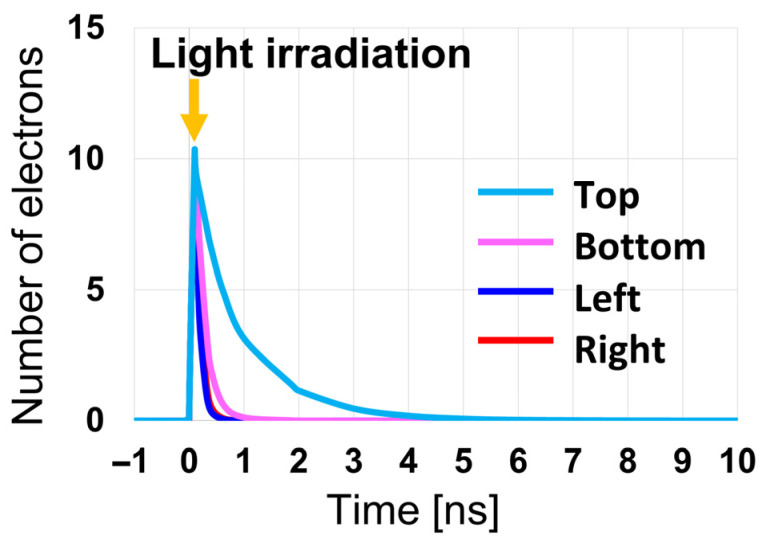
Number of electrons in the back-side photosensitive region over time after charge generation.

Figure 16 shows the time required for photo-generated charges to move from the back side of each PD to the front-side charge storage region, as obtained from device simulations. In the simulations, light was incident under conditions that generate approximately 10 electrons in the back-side photosensitive region of each pixel, and the number of electrons in this region was counted over time. The incident light positions were determined based on optical simulation results, considering the range of positions that light can reach within the pixel, and were chosen to maximize the transport distance to the charge storage region for each PD structure. As the charges are transported along the designed potential profile toward the front-side charge storage region, the number of electrons in the photosensitive region decreases over time, reaching zero once all electrons have arrived at the charge storage region. At 0 ns in Figure 16, light is incident and electrons are generated.

The charge transport simulations were performed using a small number of generated electrons (10 electrons) to evaluate the charge transfer behavior under conditions where only a limited number of carriers are instantaneously present in the photosensitive region. This condition represents a conservative case for evaluating charge transfer characteristics, because even a small loss of charge caused by local potential barriers or potential pockets can result in a significant relative error when the instantaneous carrier population is small. In contrast, when a large number of carriers are present, the relative impact of local potential nonuniformities becomes smaller compared with the overall signal fluctuation dominated by photon shot noise. By comparing the charge transfer times among the four PDs, it can be observed that the Top PD requires slightly longer time for charge transfer than the other PDs. This behavior is attributed to the influence of the floating diffusion (FD) region located directly above the Top PD, as discussed in Figure 12e,f, where the potential profile of the Top PD is affected by the FD-related potential barrier. Nevertheless, as shown in Figure 16, even for the Top PD, all photo-generated charges are successfully transferred to the charge storage region within 10 ns, confirming that fast and reliable charge transfer is achieved for all PDs in the proposed Twisted PD structure.

It should be noted, however, that the instantaneous number of carriers simultaneously present in the back-side photosensitive region is inherently limited by the rapid charge transfer to the front-side storage region. In the proposed Twisted PD, photo-generated electrons are transferred out of the photosensitive region within a few nanoseconds, as confirmed by Figure 16. Therefore, unless the illumination is extremely high, a large number of carriers cannot remain simultaneously in the photosensitive region.

This can be quantitatively estimated using the measured green sensitivity of the sensor, $S=82,000\;e^{-}/(lx\cdot s)$ (based on sensor-plane illuminance). The number of generated electrons $N_{e}$ within time t is given by $N_{e}=S\cdot E_{s}\cdot t$, where $E_{s}$ is the sensor-plane illuminance. Generating $N_{e}=10\;e^{-}$ within t=1 ns, which corresponds to the transfer time scale observed in Figure 16, requires $E_{s}\approx 1.22\times 10^{2}\;$ klx. Furthermore, assuming a Lambertian reflector with reflectance ρ=0.9, lens transmittance τ=0.9, and an F-number N=1.4, the corresponding object-plane illuminance is estimated to be approximately $1.2\times 10^{3}$ klx. For reference, this illuminance level is more than an order of magnitude higher than typical outdoor daylight conditions and is comparable to, or even exceeds, direct sunlight under clear sky conditions. This indicates that the condition in which 10 electrons are generated within a nanosecond in the photosensitive region already corresponds to a very high illumination level well beyond typical imaging conditions.

**Figure 17:**
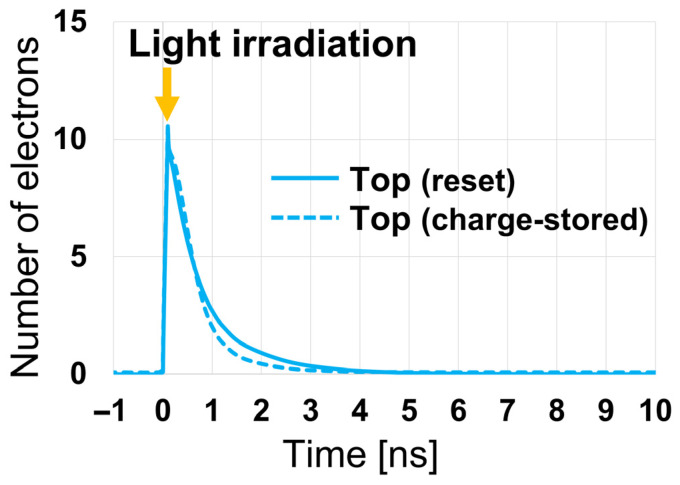
Number of electrons in the back-side photosensitive region over time after charge generation under charge-stored and reset conditions.

While the instantaneous carrier population in the photosensitive region is thus limited, the situation is different for the front-side charge storage region, where high-illumination operation can result in a large amount of accumulated charge. Such stored charge may potentially perturb the local electrostatic potential and influence subsequent charge transfer behavior. To evaluate this effect, additional charge transport simulations were performed under a charge-stored condition. Specifically, approximately 41,000 e^−^, corresponding to the sub-pixel saturation charge of one PD in the Twisted PD, was pre-stored in the Top PD charge storage region, and the same transient charge transport simulation was conducted. Figure 17 compares the time evolution of the remaining electrons in the back-side photosensitive region for the nominal (reset) condition and the charge-stored condition. As shown in Figure 17, the charge transfer time and overall transfer behavior remain essentially unchanged, confirming that fast and reliable charge transfer is maintained even under a high charge-stored condition relevant to phase-detection operation.

**Figure 18:**
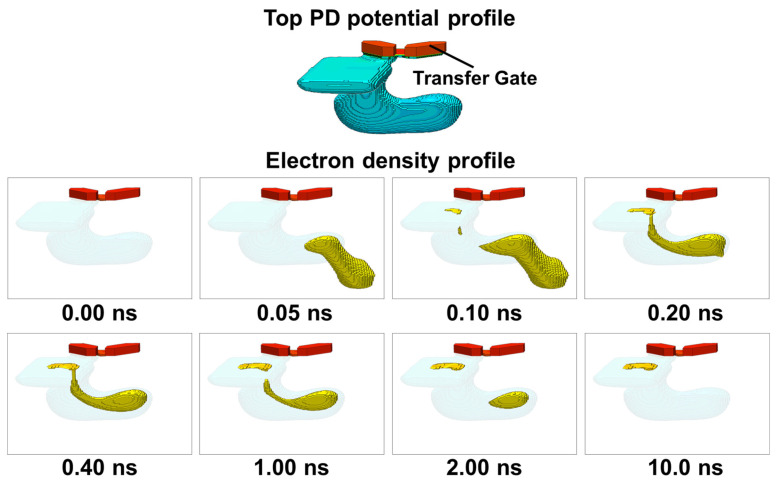
Time-dependent current density distribution for the top PD after charge generation.

Figure 18 presents the simulated charge transport in the Top PD over time. The movement of charges generated by incident light is observed at each time step, with the incident light positions determined based on the optical simulation results, as in the study for Figure 16. As confirmed in Figure 12, appropriate potential barriers are formed between adjacent PDs in the photosensitive region, preventing charge crosstalk and enabling proper carrier separation. Furthermore, as discussed in Figure 14, the p-type layer establishes a potential barrier between the front and back sides of the pixel, while the slit region provides a controlled transport path for electrons. These potential designs ensure that photo-generated charges in the back-side photosensitive region are guided through the slit toward the front side charge storage region. As shown in Figure 18, after 10 ns, all generated charges have been successfully transferred without any leakage into neighboring PDs, demonstrating rapid and well-controlled charge transport.

**Figure 19:**
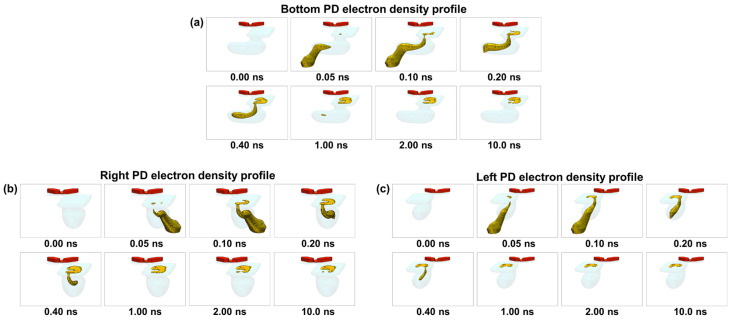
Time-dependent current density distributions for (**a**) Bottom PD, (**b**) Right PD, and (**c**) Left PD after charge generation.

While Figure 18 demonstrates the charge transport behavior for the Top PD as a representative case, it is also essential to confirm that the same transport mechanism is maintained for the other photodiodes, which have different geometries and transport directions in the Twisted PD architecture. Figure 19 shows the simulated current density over time for the Left, Right, and Bottom PDs. Although these PDs have structural configurations distinct from the Top PD, each successfully transports charges from the back-side photosensitive region to the front-side charge storage region through the designed slit pathways. Similar to the Top PD, charge transfer is completed within 10 ns for all PDs, confirming that rapid and well-controlled charge transport is achieved consistently across the entire Twisted PD structure.

**Figure 20:**
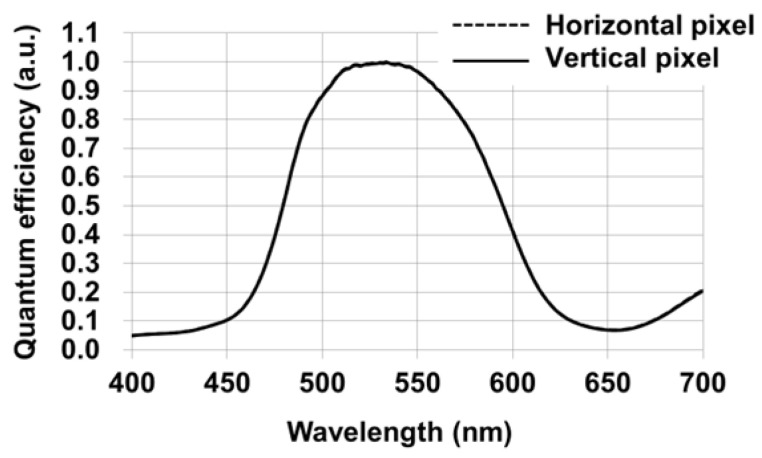
Measured quantum efficiency (QE) spectra of the horizontal and vertical pixels with green color filters. The solid line represents the horizontal pixel, and the dashed line represents the vertical pixel.

Following the confirmation of fast and reliable charge transfer for all photodiodes, we next evaluate whether the proposed Twisted PD structure maintains sufficient optical sensitivity in both pixel orientations. Since the horizontal and vertical pixels have different PD layouts and phase-detection directions, it is essential to verify that this structural difference does not introduce any sensitivity imbalance. Figure 20 shows the measured quantum efficiency (QE) spectra of the horizontal and vertical pixels, both covered with green color filters. The solid line represents the vertical pixel, while the dashed line represents the horizontal pixel. As shown in Figure 20, the QE curves of the two pixel types almost completely overlap over the entire wavelength range, indicating that there is no measurable sensitivity difference between them. This result confirms that the Twisted PD architecture preserves equivalent optical sensitivity for both horizontal and vertical pixels, despite enabling different phase-detection orientations.

**Figure 21:**
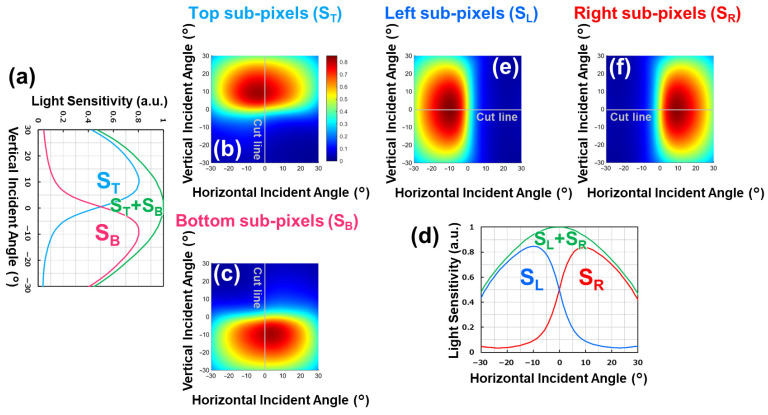
Light sensitivity dependence on incident angles. (**a**–**c**) Dependence of vertical pixels. (**d**–**f**) Dependence of horizontal pixels. Signal levels at top, bottom, left, and right sub-pixels are indicated as ST, SB, SL, and SR, respectively.

While Figure 20 confirms that both pixel types exhibit identical optical sensitivity, the key requirement for phase-detection autofocus is that each pixel generates the intended directional phase response. Therefore, we further measured the angular response characteristics of the Twisted PD pixels to experimentally validate their phase-detection functionality. Figure 21 shows the measured angular response of the Vertical and Horizontal pixels. Figure 21a,d present the angular response of the Vertical and Horizontal pixels along the vertical and horizontal directions, respectively. Figure 21b,c,e,f show the two-dimensional angular responses of the Top, Bottom, Left, and Right PDs when illuminated at varying angles in both vertical and horizontal directions. The measured responses are consistent with the charge transport simulations, confirming that charges are guided along the designed transport paths within each pixel. Based on these angular characteristics, the Vertical pixel enables phase-difference detection in the vertical direction, while the Horizontal pixel enables phase-difference detection in the horizontal direction. Unlike conventional Dual PDs, which can only provide horizontal phase-difference information, the proposed Twisted PD successfully allows the acquisition of vertical phase-difference information as well, enabling all-directional phase detection without sensitivity degradation.

**Figure 22:**
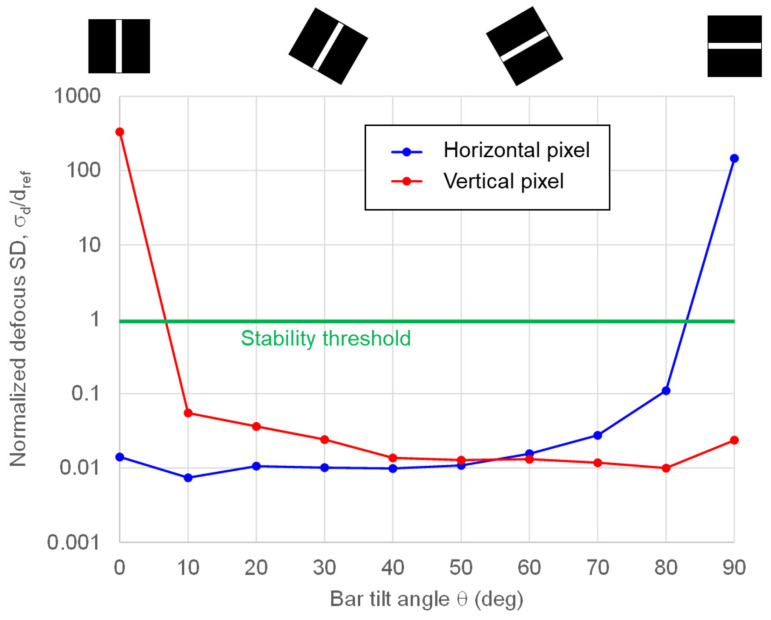
Subject-angle dependence of AF detection accuracy. The normalized standard deviation of defocus, $\sigma_{d}\left(\theta \right)/d_{ref}$ is plotted versus bar tilt angle θ (0°: vertical; 90°: horizontal) for horizontal pixels (blue) and vertical pixels (red). The green line denotes the stability threshold (=1).

While the angular response measurements in Figure 21 confirm that the Twisted PD provides directional phase-detection capability in both horizontal and vertical orientations, the ultimate requirement is whether this translates into improved autofocus robustness for subjects with arbitrary contrast directions. Therefore, we evaluated the dependence of AF detection accuracy on the subject’s edge orientation. Figure 22 shows the measured AF detection accuracy as a function of the tilt angle of a single-bar target. Accordingly, θ=0° and θ=90° are treated as extreme subject-direction cases for single-direction pixels, because the θ=0° pattern has no contrast in the vertical direction, whereas the θ=90° pattern has no contrast in the horizontal direction. Measurements were performed at EV 10.5 with an F/2.8 aperture and an integration time of 2.5 ms at the in-focus position. For each angle θ, the defocus amount d was estimated 128 times, and the standard deviation $\sigma_{d}\left(\theta \right)$ was normalized by the reference value $d_{ref}$ used as the AF stable-operation criterion. As shown in Figure 22, strong orientation dependence is observed when using only one pixel type. At θ=0° (vertical bar), AF based on horizontal pixels provides stable defocus estimates, whereas AF based on vertical pixels becomes ineffective. Conversely, at θ=90° (horizontal bar), vertical-pixel AF remains effective while horizontal-pixel AF fails. These results clearly indicate that combining horizontal and vertical pixels in a cross-type configuration enables robust phase-detection AF regardless of subject orientation, overcoming the directional limitation of conventional Dual PD pixels.

To further quantify the practical AF robustness, Table 2 summarizes the probability that the normalized defocus estimate satisfies $\left|d_{norm}\right|\leq 1$, where $d_{norm}=d/d_{ref}$. This metric corresponds to the AF success rate under the stable-operation criterion. The results confirm that AF performance strongly depends on edge orientation when only a single pixel type is used, whereas a cross-type arrangement incorporating both horizontal and vertical pixels ensures high AF success probability across all target directions.

**Figure 23:**
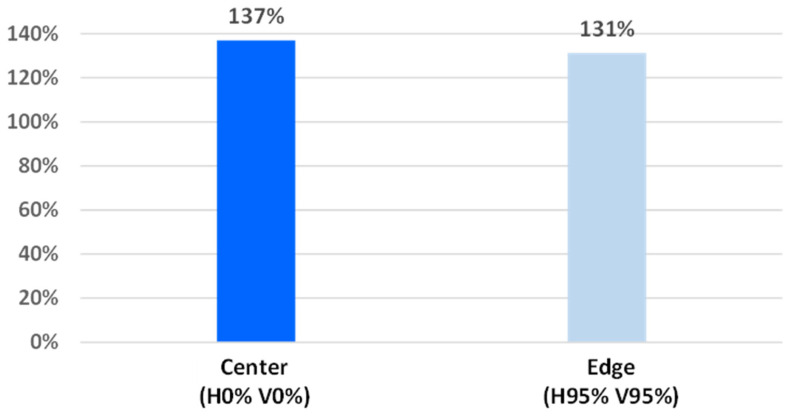
Comparison of the baseline length of the vertical pixels between the image center (H0% V0%) and a peripheral region (H95% V95%) under the same conditions as in Figure 8.

As shown in our previous study (Ref. [18]), the precision of phase-detection AF is strongly dependent on the baseline length. Ref. [18] also demonstrated AF operation up to 80% image height with a horizontal-pixel-only configuration. To evaluate center–periphery variation attributable to the sensor, Figure 23 compares the baseline length of the vertical pixels, which employ a small-slit structure with an n-type layer (using the same definition and conditions as in Figure 8), between the image center (H0% V0%) and a pe-ripheral region (H95% V95%). The baseline-length difference at the peripheral region is within 5% relative to the image center. Therefore, the position-dependent variation in AF precision attributable to the baseline length is also within approximately 5%.

**Figure 24:**
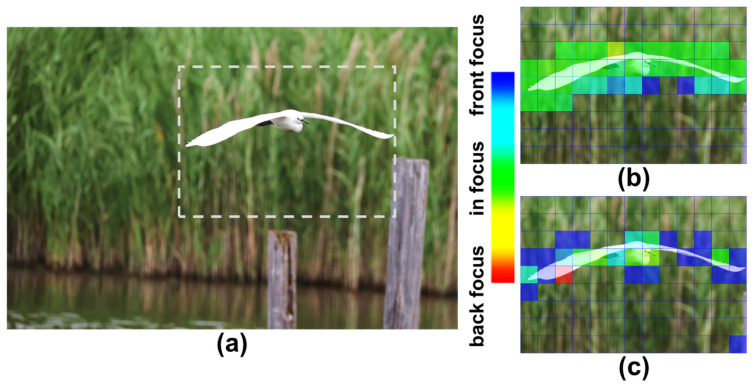
(**a**) Captured image of a flying bird taken by CMOS image sensor equipped with twisted PD and the phase detection results taken by (**b**) Vertical AF and (**c**) Horizontal AF.

Finally, to demonstrate the impact of the proposed Twisted PD in a realistic imaging scenario, phase-detection AF was evaluated using a natural subject with mixed contrast patterns. Figure 24 shows the AF results obtained from an image of a bird. Figure 24a is the captured image, while Figure 24b,c present the defocus maps obtained using Vertical AF and Horizontal AF, respectively. Regions shown in green correspond to in-focus areas, whereas blue and red indicate front-focus and back-focus regions. As observed in Figure 24b,c, AF accuracy varies depending on the dominant contrast orientation within the subject. In this example, the bird contains many horizontal line features, for which the vertical pixel provides higher phase-detection sensitivity, resulting in improved AF accuracy with Vertical AF. Because the proposed Twisted PD integrates pixels optimized for both horizontal and vertical contrast patterns, accurate autofocus can be achieved for a wide variety of real-world subjects. This demonstrates the advantage of the Twisted PD architecture in enabling all-directional and robust phase-detection AF beyond the limitations of conventional Dual PD designs.

Table 3 summarizes the comparison of key performance metrics with previous studies. As shown in the table, the proposed Twisted PD achieves all-directional phase detection while maintaining high optical performance. In particular, a high green sensitivity of 82,000 e^−^/lux/s is realized through the optimized pixel optical structure incorporating micro-lenses and inner micro-lenses, demonstrating that the added phase-detection functionality does not compromise light collection efficiency. Moreover, the 100% density of AF pixels significantly improves autofocus robustness under low-illumination conditions, where AF errors become prominent, enabling a minimum AF illuminance level as low as 0.007 lux. These results highlight that the Twisted PD provides a practical solution to extend phase-detection capability beyond conventional Dual PD approaches, achieving both high sensitivity and robust all-directional AF performance. Based on these experimental and simulation results, the conclusions of this work are summarized in the next section.

## 4. Conclusions

Autofocus (AF) is one of the most essential functions in CMOS image sensors (CIS), and achieving both high speed and high accuracy over a wide range of subjects and shooting conditions remains a key challenge. In this work, we demonstrated that the proposed Twisted Photodiode (PD) structure enables all-directional phase detection while preserving pixel-level symmetry, thereby addressing an inherent structural trade-off in PDAF pixel design. Although the Twisted PD introduces a more complex three-dimensional charge transport path compared with conventional Dual PD structures, reliable and fast charge transfer was ensured through an appropriate electrostatic design. The combination of the p-type separation layer and the slit structure effectively guides photo-generated charges toward the front-side charge storage region, while the partially introduced n-type layer beneath the p-type region enables proper potential formation even in the back-side photosensitive region. Device simulations confirmed that these structural elements collectively establish a monotonic electrostatic potential gradient along the three-dimensional transfer path, allowing rapid and stable charge transfer without degradation. Imaging experiments using a CIS implementing the Twisted PD further validated the practical effectiveness of the proposed structural concept. Vertical pixels exhibited improved AF accuracy for subjects dominated by horizontal line patterns, while horizontal pixels retained high performance for vertical patterns. By integrating both pixel orientations within a single pixel array, the Twisted PD achieves robust all-directional phase detection across a broad range of subject textures and orientations, without compromising pixel uniformity or charge transfer characteristics. Taken together, these results demonstrate that the Twisted PD provides not merely an engineering implementation, but a structure-driven design approach that reconciles all-directional phase-detection capability with pixel structural symmetry. The presented charge transfer analysis serves as a physics-based validation of this concept, confirming its suitability for high-performance PDAF image sensors.

For future implementations, several design considerations related to three-dimensional pixel configuration and scalability merit further investigation. Because the separation direction between adjacent PDs differs between the front-side and back-side regions, carriers generated deep in silicon by long-wavelength light could potentially experience increased crosstalk due to spatial overlap along the depth direction. In the present design, this effect is mitigated by placing vertical pixels only at the Gb positions in the Bayer array—where long-wavelength light is largely attenuated—and by optimizing the depth of the p-type separation layer. Furthermore, while sufficient margin exists for the slit geometry in the current sensor with a pixel pitch of 6 µm, continued pixel miniaturization will impose tighter constraints on slit dimensions. Ongoing optimization of slit design will therefore be essential to maintain reliable charge transfer characteristics in scaled Twisted PD structures.

## Figures and Tables

**Table 1:** Qualitative comparison of various PDAF pixel architectures. The qualitative performance levels are visually highlighted to facilitate comparison among different PDAF pixel architectures.

	Conventional PD	Quad PD	Slanted PD	Rotated PD	Twisted PD (This Work)
**Plan view**	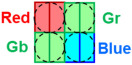	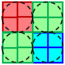	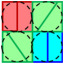	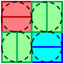	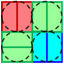
**Pixel structure**	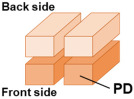	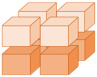	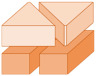	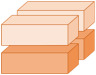	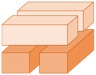
**PDAF accuracy**	High	High	Moderate	High	High
**Vertical PDAF**	Limited	Supported	Partially supported	Supported	Supported
**DRN**	Low	Increased	Low	Moderate	Low
**FWC**	Large	Reduced	Large	Moderate	Large
**H/V pixel mismatch**	Small	Small	Small	Large	Small
**Readout time**	Short	Long	Short	Short	Short
**Data processing**	Simple	Complex	Complex	Simple	Simple

**Table 2:** AF success rate versus bar tilt angle. For each angle (N=128), the normalized defocus is defined as $d_{norm}=d/d_{ref}$. The AF success rate is the fraction of trials with $\left|d_{norm}\right|\leq 1$.

Angle	0°	10°	20°	30°	40°	50°	60°	70°	80°	90°
Horizontal	100%	100%	100%	100%	100%	100%	100%	100%	100%	10%
Vertical	0%	100%	100%	100%	100%	100%	100%	100%	100%	100%

**Table 3:** Comparison of sensor characteristics.

	This Work	H. Kim et al. (2023) [6]	T. Jung et al. (2022) [5]	Y. Sawai et al. (2021) [19]	E.S. Shim et al. (2021) [4]	T. Okawa et al. (2019) [3]	M. Kobayashi et al. (2015) [1]
Pixel size	**6.0 µm**	1.28 µm	1.0 µm	5.6 µm	1.4 µm	1.6 µm	6.4 µm
AF direction	**Horizontal/Vertical**	Horizontal/Vertical	Horizontal/Vertical	Horizontal	Horizontal/Vertical	Horizontal	Horizontal
Full well capacity	**121,000 e^−^**	20,000 e^−^	10,000 e^−^	100,000 e^−^	10,000 e^−^	―	40,000 e^−^
Sub-pixel saturation	**41,000 e^−^**	―	―	―	―	―	―
Sensitivity †	**82,000 e^−^/lux/s**	6480 e^−^/lux/s	3430 e^−^/lux/s	―	7000 e^−^/lux/s	―	78,000 e^−^/lx/s
Conversion gain	**125/30.2/15.1 µV/e^−^**	―	―	152/24.5/11.5 µV/e^−^	―	―	70 µV/e^−^
Random noise	**2.2/8.3/16.7 e^−^rms**	0.98 e^−^rms	2.0 e^−^rms	5/-/30 e^−^rms	2.0 e^−^rms	―	2.5 e^−^rms
Dynamic range †††	**95 dB**	86 dB ††	74 dB ††	85 dB	74 dB ††	―	84 dB ††
Image lag	**<1.0 e^−^**	<1.0 e^−^	<1.0 e^−^	<1.0 e^−^	<1.0 e^−^	―	―
AF density	**100%**	―	―	―	―	100%	100%
Minimum AF level	**0.007 lux**	―	―	―	―	1 lux	―

† Measured based on different color temperature light source. †† Calculated based on disclosed information. ††† Calculated with random noise and FWC.

## Data Availability

The data presented in this study are available on request from the corresponding author. The data are not publicly available due to the confidentiality of the corporate activity.
